# Decellularized dermis allograft for the treatment of venous leg ulceration: the DAVE RCT

**DOI:** 10.1093/bjs/znae330

**Published:** 2025-02-17

**Authors:** Sarah Onida, Matthew Tan, Valeria Balan, Francine Heatley, Sarrah Peerbux, Layla Bolton-Saghdaoui, Tristan Lane, David Epstein, Manjit Gohel, John Norrie, Robert Lee, Richard Lomas, Alun H Davies

**Affiliations:** Section of Vascular Surgery, Department of Surgery and Cancer, Imperial College London, London, UK; Section of Vascular Surgery, Department of Surgery and Cancer, Imperial College London, London, UK; Section of Vascular Surgery, Department of Surgery and Cancer, Imperial College London, London, UK; Section of Vascular Surgery, Department of Surgery and Cancer, Imperial College London, London, UK; Section of Vascular Surgery, Department of Surgery and Cancer, Imperial College London, London, UK; Section of Vascular Surgery, Department of Surgery and Cancer, Imperial College London, London, UK; Section of Vascular Surgery, Department of Surgery and Cancer, Imperial College London, London, UK; Cambridge Vascular Unit, Addenbrooke’s Hospital, Cambridge University Hospitals NHS Foundation Trust, Cambridge, UK; Department of Applied Economics, University of Granada, Granada, Spain; Cambridge Vascular Unit, Addenbrooke’s Hospital, Cambridge University Hospitals NHS Foundation Trust, Cambridge, UK; Edinburgh Clinical Trials Unit, University of Edinburgh, Edinburgh, UK; Edinburgh Clinical Trials Unit, University of Edinburgh, Edinburgh, UK; Tissue and Eye Services, National Health Service Blood and Transplant, Bristol, UK; Section of Vascular Surgery, Department of Surgery and Cancer, Imperial College London, London, UK

## Abstract

**Background:**

Venous leg ulcers (VLUs) cause significant impairment to patients’ quality of life (QoL) and up to 30% do not heal at 6 months. Decellularized dermis (DCD) allografts have been shown to be effective in improving healing rates of diabetic foot ulcers in RCTs. The DAVE RCT aimed to determine whether DCD is an effective, safe, and cost-effective treatment adjunct for VLUs.

**Methods:**

This was a multicentre RCT performed in the UK. Patients with lower limb ulcers ≥18 years with VLU, venous incompetence on duplex ultrasound, an ankle : brachial pressure index ≥ 0.8 and an index VLU present for at least 3 months and ≥2 cm^2^ in size were included. Patients were randomized to either the intervention (DCD graft and standard of care) or control arm (standard of care alone). The primary outcome was the proportion of patients with a healed index ulcer at 12 weeks.

**Results:**

From October 2019 to October 2022, 36 and 35 participants were randomized into the intervention and control arms respectively. Patient characteristics at baseline were similar between groups. Healing was recorded in 5.7% of the intervention group and 15.2% in the control group (OR 0.34, 95% c.i. 0.03 to 2.31). There were no significant differences in the secondary outcomes including the percentage change in ulcer area, time to healing, recurrence rates, and QoL. There were five serious adverse events attributed to DCD application. Early trial termination was advised after the interim data analysis due to a lower-than-expected primary outcome rate (11.3%).

**Conclusions:**

Decellularized dermis grafts did not improve healing rates of venous leg ulcers, although the trial was terminated early due to poor healing rates in both the intervention and control arms. Important clinical benefits or harms of decellularized dermis grafts could not be excluded due to the small sample size.

**Trial registration:**

ISRCTN 21541209

## Teaser

Venous leg ulcer (VLU) healing remains a challenge despite standard of care and human decellularized dermis allograft (DCD) may be an effective adjunct for chronic VLU care. This RCT explores the use of DCD in chronic VLUs as an adjunct to standard of care. It shows that DCD does not seem to be an effective treatment adjunct at improving healing.

## Introduction

Venous leg ulcers (VLUs) are lower-limb wounds caused by an inadequately functioning venous system. They affect around 1% of the general population, increasing to 4% among individuals aged 65 and older^1^. VLUs can be painful, leading to decreased mobility, sleep disturbance, and require frequent dressing changes, which can be extremely uncomfortable. The negative effects on health-related quality of life (HRQoL) can be as severe as those observed in other chronic conditions, including heart failure and chronic obstructive pulmonary disease^2^.

Financially, VLUs impose a substantial burden on the healthcare system. With a high prevalence of VLU patients in the community, district nurses spend up to 50% of their time caring for chronic wounds, with approximately 70% of wounds being of venous origin^3,4^. Moreover, there is a considerable recurrence risk, with up to 20% of VLUs recurring at 1 year and up to 48% at 5 years, necessitating further treatment^5,6^. In Western societies, VLU accounts for up to 2% of the annual healthcare budget, with the cost reaching approximately £3.2 billion in the UK in 2017^7^. As the population ages, this burden is projected to escalate^8^.

In national and international guidelines, it is typically recommended that VLU management includes wound care, compression therapy, and endovenous ablation. To accelerate and facilitate VLU healing, these guidelines also recommend skin grafting as an adjunctive treatment^9–11^. Multiple different types may be used, including autografts, allografts, or tissue-engineered skin. A Cochrane Systematic Review identified that tissue-engineered skin, when used alongside compression therapy, increased VLU healing, but concluded that there was insufficient evidence to determine the effectiveness of other graft materials^12^.

Human decellularized dermis (DCD) is produced from cadaveric skin that undergoes a decellularization process to eliminate epidermal and dermal cells while maintaining the dermal structure^13^. This results in an immunologically inert scaffold that is incorporated into the wound by facilitating cellular repopulation and tissue revascularization. One significant benefit is its versatility in application. It can be applied to the wound using local anaesthesia, either through tissue staples or sutures, or even without anaesthesia using tissue glue, eliminating the need for hospital admission and general anaesthesia for the procedure.

The aim of this study was to evaluate whether DCD in addition to standard care improved the outcomes of VLUs compared to standard care alone.

## Methods

### Study design

This was a prospective, randomized, open label, pragmatic trial^14^ conducted across 17 centres in the UK. Planned study activities lasted up to 12 months for each participant. The study received approval from the UK National Research Ethics Service (Bloomsbury Research Ethics Committee) on 6 February 2019 (REC reference number: 19/LO/1271). The design and execution specifics of the trial can be found in the previously published protocol^15^. The trial was registered on the ISRCTN registry (registry number: ISRCTN 21541209). All participants provided written informed consent, and the trial was overseen by a trial steering committee and an independent data monitoring committee (DMC). Funding was provided by the JP Moulton Charitable Trust after open competition for grant funding.

### Power calculations

At 90% power and 5% level of significance, 196 patients were required. This assumed a healing rate of 30% and 55% in the control and intervention groups respectively (absolute difference of 25% estimated based on previously published literature on DCD in diabetic and venous ulceration^16–18^) and allowed a 10% loss to follow-up.

### Inclusion criteria

- Able to provide informed written consent
- ≥18 years old
- Leg ulcer (ulcer proximal to malleoli) with documented venous incompetence on Duplex ultrasound or handheld continuous wave Doppler to allow for recruitment in non-tertiary centres which do not have access to Duplex ultrasound
- Ulcer area ≥2 cm^2^
- Ulcer present for >3 months
- Ankle : brachial pressure index ≥0.8

### Exclusion criteria

- Foot ulcer (ulcer distal to malleoli)
- Diagnosis of sickle cell disease
- Unable to receive one or more of the randomized treatment strategies for any reason at the discretion of the attending clinical team
- Clinical evidence of infection (for example erythema, cellulitis, systemically unwell)
- Usage of systemic/topical growth factors within previous 30 days
- Previous history of inability to tolerate compression therapy

### Randomization and masking

**Fig. 1:**
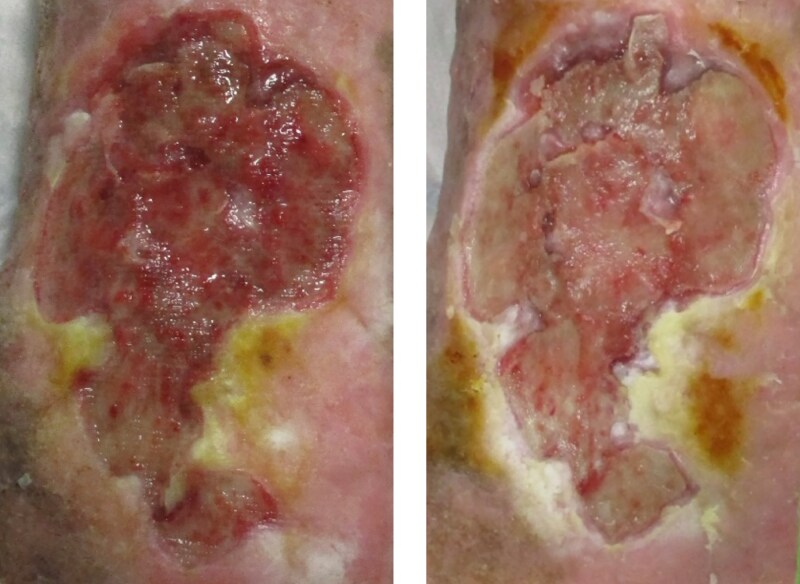
An example ulcer pre-graft application (left), and the DCD graft which is still visible a week after application (right)

Participants were randomized 1 : 1 to either the intervention or control arms using an automated system that is linked to the electronic case report form (eCRF) through the Edinburgh Clinical Trials Unit. A minimization algorithm was employed, considering the centre, index ulcer size, and ulcer duration, including an element of randomness to enhance unpredictability. Due to the graft being visible for a certain duration after application, it was not feasible to conceal the treatment strategy from participants or the research/clinical teams (*Fig. 1*).

### Interventions

All participants underwent wound cleaning and debridement. In the control arm, it was followed by standard compression therapy (multilayer elastic compression bandaging or stockings). In the intervention arm, it was directly followed by DCD allograft application. Before being applied, the product was soaked in a bowl of sterile saline solution for 15 min. The graft was placed directly onto the cleaned wound bed, with the epidermal side facing upwards. It was secured using liquid skin adhesive, sutures, or staples, depending on clinician choice. A non-adhesive, non-absorbent, non-medicated primary dressing was applied, followed by appropriate bolster/secondary dressings and, finally, standard compression therapy.

Participants were allowed to undergo other interventions including superficial venous treatment and surgical wound debridement. The indication for and intervention type were left to the treating clinician's discretion, and these were recorded in the eCRF.

### Outcomes

The primary outcome was the proportion of participants with a healed index ulcer (if multiple present, the largest ulcer) at 12 weeks after randomization, defined as total re-epithelialization, excluding any scabs or eschar, and without the need for a dressing. This outcome was verified through a blinded photographic evaluation of the healing process by two blinded assessors. Starting from the 6-week point, biweekly telephone calls were initiated to assess ulcer healing progress to guarantee a precise assessment of the time required for healing. When an ulcer was determined to be healed within the study’s duration, the patient was required to attend a visit specifically for photographic verification. Whenever feasible, this visit was conducted within a 7-day time frame. Patients were classified as reaching the primary outcome of a healed index ulcer at 12 weeks after randomization if they had a verified ulcer healing date within 12 weeks of randomization with no recurrence or recurrence more than 12 weeks after randomization, or if the index ulcer recurred and healed again within 12 weeks of randomization. Assessment of this outcome was carried out by two impartial blinded clinical assessors who were well-trained in evaluating wound healing.

Secondary outcome measures included time to index ulcer healing, the percentage change in index ulcer area at 12 weeks, the proportion of participants with a healed index ulcer at 12 months, the proportion of participants whose index ulcer healed for whom an ulcer recurred at the index site within 12 months, HRQoL analysis (Charing Cross Venous Ulcer Questionnaire [CCVUQ], EuroQol-5 Dimension-5 Level [EQ-5D-5L]), and cost-effectiveness analysis, which will be published separately.

### Statistical analysis

A modified intention-to-treat analysis was performed. Due to the study's early termination, the original statistical analysis plan was revised to reflect the underpowered nature of the trial, switching from hypothesis testing to descriptive statistics with measures of precision (for example 95% confidence intervals). Due to the small number of events, exact logistic regression was used for any logistical regression analysis. Additionally, planned sensitivity analysis based on missing data patterns was not carried out. No per-protocol analyses or causal models were conducted to adjust for randomized intervention adherence.

The intervention effect on the odds of the primary outcome was assessed using an exact logistic regression model, with no adjustment for study site as a random effect, or index ulcer size and duration at baseline as fixed effects.

Time from randomization to index ulcer healing was compared by graph with Kaplan–Meier estimates of cumulative healing over time in each treatment arm. Difference in mean percentage change in index ulcer area at 12 weeks compared to baseline was assessed using a linear model. Exact logistic regression models were used to assess the participant proportion in each arm with a healed index ulcer at 12 months, and in participants with a healed index ulcer, the proportion with ulcer recurrence at the index site within 12 months of randomization.

The effect of the intervention on HRQoL was assessed using a repeated-measures linear mixed model, with an unstructured covariance structure for the repeated measurements over time, including terms for treatment arm, time (12 weeks, 6 months, 12 months), treatment arm by time interaction, and adjusting for HRQoL at baseline as a fixed effect using a linear term.

Adverse events and serious adverse events and suspected unexpected serious adverse reactions are summarized by study arm.

### Role of the funder

This study was funded by the JP Moulton Charitable Trust (project number 14/140/61). The funders had no role in study design, data collection, analysis, and interpretation, or writing of the report.

## Results

### Study screening and enrolment

**Fig. 2:**
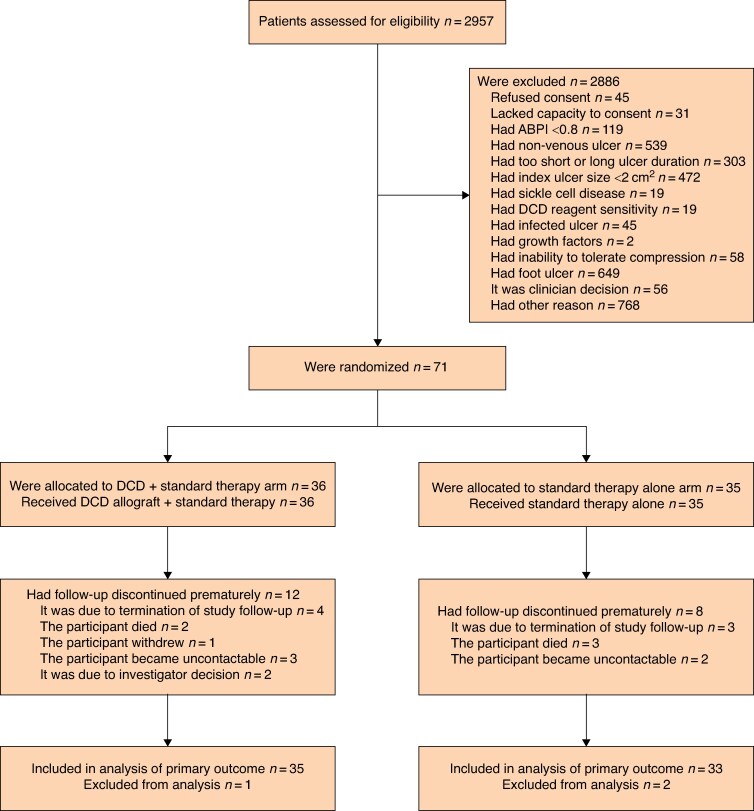
CONSORT diagram

Of 2957 individuals assessed, 2886 were excluded (*Fig. 2*). The largest group was excluded under the ‘Other’ criterion (*n* = 768), with the majority being excluded for only having varicose veins with recently healed ulcers. With respect to specific criteria, participants were excluded due to having a foot ulcer (*n* = 649), a non-venous ulcer (*n* = 539), and having an index ulcer size <2 cm^2^ (*n* = 472).

A total of 303 participants were deemed ineligible based on ulcer duration. At the start of this trial, the relevant inclusion criterion was such that patients with an index ulcer duration of <6 months or >24 months were ineligible for the trial. To facilitate recruitment, this criterion was changed twice, with the first amendment in February 2020 to only exclude patients with an index ulcer duration of <6 months, and again in January 2022 to exclude patients with an index ulcer duration of <3 months.

### Participant recruitment and follow-up

The study was terminated early following an interim data analysis at an independent DMC meeting on 14 September 2022. At that time, 67 participants were randomized, with only seven having recorded the primary outcome. This represented an 11% primary outcome rate, which was lower than the expected average of 43% that was used for power calculation. Given the inability to detect a clinically meaningful difference in the primary outcome even if the target sample size was reached, further recruitment was terminated.

A total of 71 participants were recruited from 11 centres (69.0% male, 68.0 ± 14.9 years old). Thirty-six and 35 participants were allocated to the intervention and control groups respectively. At randomization, there were no differences between the demographic and clinical characteristics of the groups. The overall median index ulcer area was 10.6 cm^2^ (i.q.r. 4.6 to 24.1) and median ulcer age was 15 months (i.q.r. 8 to 48) (*Table 1*, *Table S1*). Fifty-one participants completed the study and 20 ended their participation prematurely (*Fig. 2*). The final 12-month patient follow-up examination was completed on 10 April 2023.

**Table 1:** Baseline characteristics of randomized patients

	DCD + standard therapy (*n* = 36)	Standard therapy alone (*n* = 35)	Overall (*n* = 71)
Age (years), mean ± s.d.	67.6 ± 15.5	67.5 ± 14.4	67.5 ± 14.9
**Sex, no. (%)**			
Male	23 (64)	26 (74)	49 (69)
Female	13 (36)	9 (26)	22 (31)
**Ethnicity, no. (%)**			
White	32 (89)	29 (83)	61 (86)
Asian or Asian British	2 (6)	3 (9)	5 (7)
Black or Black British	2 (6)	3 (9)	5 (7)
BMI (kg/m^2^), mean ± s.d.	33.9 ± 11.2	32.2 ± 7.9	33.1 ± 9.7
**Smoking status, no. (%)**			
Current	4 (11)	3 (9)	7 (10)
Former	15 (42)	16 (46)	31 (44)
Never	17 (47)	16 (46)	33 (46)
Ulcer size (cm^2^), median (i.q.r.)	11.1 (5.4–32.8)	8.0 (4.2–20.4)	10.6 (4.6–24.1)
Ulcer age (months), median (i.q.r.)	20 (9–54)	14 (8–48)	15 (8–48)
Diabetes, no. (%)	12 (33)	4 (11)	16 (23)
History of deep venous thrombosis, no. (%)	12 (33)	11 (31)	23 (32)
Previous leg ulceration in the reference leg, no. (%)	23 (64)	19 (54)	42 (59)

### Primary outcome

Participants were included in the primary outcome analysis if they had at least one follow-up for ulcer healing at 12 weeks or more after randomization. From the intervention and control groups, 35 and 33 participants were included respectively, with three participants excluded as they did not attend any follow-up visits, leading to a modified intention-to-treat analysis. There were no differences in primary outcome with odds ratio of 0.34 (95% c.i., 0.03 to 2.31) observed between the intervention (5.7%, *n* = 2) and control (15.2%, *n* = 5) groups.

### Secondary outcomes

**Fig. 3:**
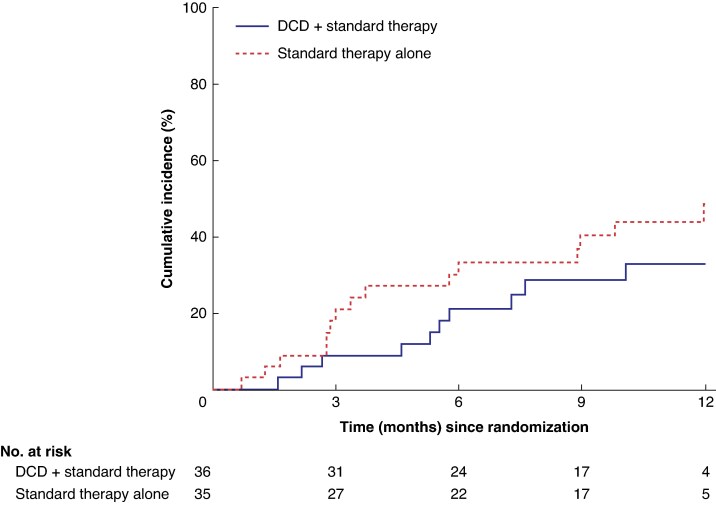
Cumulative incidence of healed index ulcer and healed index ulcer within 12 months of randomization

A Cox proportional hazards model showed no difference in the cumulative incidence of healed index ulcers between the two groups after 12 months of randomization. The unadjusted hazard ratio was 0.64 (95% c.i., 0.29 to 1.42; *Fig. 3*). This was also reflected in the analysis of 51 participants who completed the study, with follow-up for ulcer healing at 12 months, with no differences in the proportion of participants who had a healed index ulcer, with an unadjusted OR of 0.63 (95% c.i., 0.17 to 2.24; *Table S2*).

Percentage change in index ulcer area at 12 weeks after randomization was analysed for 66 participants who had index ulcer area measurements using planimetry tracings at baseline and at least once during the follow-up period. There was no significant difference between groups, with an unadjusted difference in mean percentage change of −1.3 cm^2^ (95% c.i., −30.8 to 28.2; *Table S3*).

Ulcer recurrence rate was analysed for 22 participants who had a healed index ulcer and either recurrence within 12 months of randomization or follow-up to 12 months without recurrence. There were no differences between groups (unadjusted OR 4.39, 95% c.i. 0.29 to 269.5). It must be noted that due to the small number of participants with a healed index ulcer, the actual recurrence numbers in each group were small (three participants and one participant in the intervention and control groups respectively; *Table S4*).

There were no statistically significant differences between groups when measured using both generic and disease-specific HRQoL instruments at 12 weeks, 6 months, and 12 months (*Table S5*). When considering generic HRQoL, the intervention group showed HRQoL deterioration during the trial while the control group experienced an improvement. However, when comparing the mean EQ-5D health index scores at each timepoint, this revealed non-significant difference in means of 0.097 (95% c.i. −0.013 to 0.207), −0.013 (95% c.i. −0.145 to 0.119) and −0.105 (95% c.i. −0.214 to 0.003). This was also seen in the EQ-5D health scale results (*Table S6*).

Both groups showed an improvement in disease-specific HRQoL, with a larger improvement in CCVUQ absolute score experienced by the control group (*Table S5*). However, non-significant differences in mean scores were seen at each timepoint of measurement (*Table S6*).

Serious adverse events (SAEs; *n* = 22) were reported, with 17 events being classified as not related and five as related to the DCD (*Table S7*). Thirteen and nine SAEs were reported in the intervention and control groups respectively. Non-serious adverse events related to skin graft or leg ulcer (*n* = 37) were reported with the most common adverse events being wound or graft infection (*n* = 18) and pain (*n* = 7) (*Table S8*).

## Discussion

The DAVE trial investigated DCD allograft as an adjunctive treatment in VLU patients, a gap in the literature identified by a Cochrane review. Previous trials utilizing the DCD allograft were performed in diabetic foot ulceration, demonstrating improvements in wound healing^17,19–22^, with smaller cohort studies suggesting efficacy in VLU patients as well^16^.

The DAVE trial has not been able to rule out substantial harm or benefit of DCD allograft as an effective treatment due to the small sample size consequent on early closure due to a low event rate. At the primary endpoint, only 5.7% of the ulcers in the intervention arm were healed. In the control arm, only 15.2% of ulcers were healed, much lower than the healing rates of up to 30% that have been reported^23^. The overall poor healing rate observed may be related to the COVID-19 pandemic, with a decline in VLU referrals and a reduction in the delivery of routine care. A nationwide study showed significant delays in providing superficial venous interventions for patients with chronic venous disease^24^. Another study revealed decreased dressing change frequency from a mean of once every 11 days to 21 days between 2020 and 2021^25^. The rate of attendance for dressing changes or compression application was not documented as part of this trial and should be a parameter for inclusion in future studies. This same study also concluded that VLU healing rates declined by 16% and 42% in 2020 and 2021 respectively^25^. Further epidemiological studies may be required to determine if VLU healing rates have indeed deteriorated.

Issues with graft application and retention may have contributed significantly to the intervention arm's poor healing rates. Several sites reported accidental allograft removal during regular dressing changes in the community. This is despite information leaflets being provided to participants to be given to the community care providers during visits and education sessions being offered to the community care providers when required. In two cases, the allograft was debrided as it was mistakenly identified as slough during wound care visits. This necessitated repeat wound bed preparation and allograft application, delaying VLU healing. Together with a consistent wound bed preparation, proper allograft fixation is key to integration, which can be difficult given that VLUs are usually near or over the ankle. These issues in the community and with graft application further suggest that it will not be a modality that can be easily adopted outside of tertiary wound care or vascular centres.

Enrolment was hampered by a high screen failure rate, with multiple candidates found to have recently healed ulcers. This could partially be due to the significant delays in referrals from primary care, reflected in the many studies that have reported long referral times globally^26,27^, resulting in patients with VLUs healing prior to being reviewed in a secondary or tertiary healthcare setting in which this trial was mostly delivered. These referral delays have been further affected by the COVID-19 pandemic as discussed above.

The results were found to be underpowered due to the small numbers of healers in both groups during the interim data analysis. For a study to have 90% power at a 5% level of significance to detect a change from 15% to 29% (which would correspond to the observed upper 95% confidence interval on the odds ratio of 2.34 seen in this study) for the same primary outcome of proportion with a healed index ulcer at 12 weeks would require 400 participants (allowing 10% loss to follow-up). Whether such a trial is feasible (or possibly a smaller trial, if the 71 randomized here could be statistically incorporated into the new trial, for example through an individual patient data meta-analysis, or through a Bayesian design) would rely on gaining funding and learning lessons on recruitment offered from this current DAVE trial.

The DAVE trial has shown that DCD allograft as an adjunctive treatment for VLUs may not be effective at improving healing rates in the NHS. There were no other benefits of DCD identified, although these findings are limited due to the underpowered nature of the trial.

## Supplementary Material

znae330_Supplementary_Data

## Data Availability

The data underlying this article will be shared on reasonable request to the corresponding author.

## Supplementary material

Supplementary  material is available at *BJS* online.

## Data access statement

The trial data will be available on request to the chief investigator (Professor Alun Davies).
