# Supplementary material for: Decellularized dermis allograft for the treatment of venous leg ulceration: the DAVE RCT
Source: Br J Surg. 2025 Feb 17;112(2):znae330. doi: 10.1093/bjs/znae330 (PMC11831364; doi:10.1093/bjs/znae330)

**Title: Decellularised Dermis Allograft for the Treatment of Venous Leg Ulceration: The DAVE RCT**

**Authors:** Sarah Onida*^1^ MBBS BSc PhD FRCS, Matthew Tan*^1^ MBBS BSc MRCS, Valeria Balan^1^ BSc MPH, Francine Heatley^1^ BSc PhD, Sarrah Peerbux^1^ BA MSc, Layla Bolton-Saghdaoui^1^ BSc, Tristan Lane^1,2^ MBBS BSc PhD FRCS, David Epstein^3^ MSc PhD, Manjit Gohel^2^ MD FRCS, John Norrie^4^ BSc MSc PhD, Robert Lee^4^ PhD, Richard Lomas^5^ PhD, Alun H Davies^1^ MA DM DSc FRCS FEBVS FACPh

^1^: Section of Vascular Surgery, Department of Surgery and Cancer, Imperial College London, London, United Kingdom

^2^: Cambridge Vascular Unit, Addenbrooke’s Hospital, Cambridge University Hospitals NHS Foundation Trust, Cambridge, United Kingdom

^3^: Department of Applied Economics, University of Granada, Granada, Spain

^4^: Edinburgh Clinical Trials Unit, University of Edinburgh, Edinburgh, United Kingdom

^5^: National Health Service Blood and Transplant, Bristol, United Kingdom

**Corresponding author.** Professor Alun H Davies

Address: Room 4E04, 4th Floor East Wing, Charing Cross Hospital, Fulham Palace Road, Hammersmith, London W6 8RF

**Supplementary Materials - Index**

| **Supplementary Figures and Tables** |  |
| --- | --- |
| Table S1. Baseline characteristics of randomised patients | *page 2* |
| Table S2. Healed index ulcer at 12 months after randomisation | *page 5* |
| Table S3. Percentage change in index ulcer area at 12 weeks after randomisation | *page 5* |
| Table S4. Ulcer recurrence at index site within 12 months of randomisation | *page 5* |
| Table S5. Summary statistics for generic and disease-specific health-related quality of life measures | *page 6* |
| Table S6. Difference in means at each timepoint for generic and disease-specific health-related quality of life measures | *page 7* |
| Table S7. Serious and non-serious adverse events | *page 8* |
| Table S8. Non-serious adverse events related to skin graft or leg ulcer | *page 8* |
| **References** | *page 9* |
|  |  |

**Supplementary Figures and Tables**

**Table S1. Baseline characteristics of randomised patients**

|  | **DCD + Standard Therapy (n = 36)** | **Standard Therapy Alone (n = 35)** | **Overall (n = 71)** |
| --- | --- | --- | --- |
| **Demographics** | | | |
| Age (years) – mean +/- SD | 67.6 +/- 15.5 | 67.5 +/- 14.4 | 67.5 +/- 14.9 |
| Sex – no. (%)  Male  Female | 23 (64)  13 (36) | 26 (74)  9 (26) | 49 (69)  22 (31) |
| Ethnicity – no. (%)  White  Asian or Asian British  Black or Black British | 32 (89)  2 (6)  2 (6) | 29 (83)  3 (9)  3 (9) | 61 (86)  5 (7)  5 (7) |
| Body mass index (kg/m^2^) – mean +/- SD | 33.9 +/- 11.2 | 32.2 +/- 7.9 | 33.1 +/- 9.7 |
| **Ulceration Characteristics** | | | |
| Ulcer size (cm^2^) – median (IQR) | 11.1 (5.4-32.8) | 8.0 (4.2-20.4) | 10.6 (4.6-24.1) |
| Ulcer age (months) – median (IQR) | 20 (9-54) | 14 (8-48) | 15 (8-48) |
| Previous skin graft to index ulcer – no. (%) | 7 (19) | 3 (9) | 10 (14) |
| Type of graft – no. (%)  Autograft  DCD allograft | 4 (57)  4 (57) | 2 (67)  1 (33) | 6 (60)  5 (50) |
| Previous leg ulceration in the reference leg – number. (%) | 23 (64) | 19 (54) | 42 (59) |
| Previous leg ulceration in the non-reference leg – no. (%) | 18 (51) | 10 (29) | 28 (40) |
| Current leg ulceration in the non-reference leg – no. (%) | 8 (22) | 13 (37) | 21 (30) |
| **Lifestyle Characteristics** | | | |
| Currently employed – no. (%)  Full time  Part time  Unemployed  Retired | 7 (19)  1 (3)  6 (17)  22 (61) | 8 (23)  1 (3)  6 (17)  20 (57) | 15 (21)  2 (3)  12 (17)  42 (59) |
| Smoking Status – no. (%)  Current  Former  Never | 4 (11)  15 (42)  17 (47) | 3 (9)  16 (46)  16 (46) | 7 (10)  31 (44)  33 (46) |
| If current smoker: Average number of cigarettes/pipes per day  <5  5-9  10-14  15-24  >=25 | 1 (25)  1 (25)  0 (0)  2 (50)  0 (0) | 1 (33)  1 (33)  1 (33)  0 (0)  0 (0) | 2 (29)  2 (29)  1 (14)  2 (29)  0 (0) |
| If former smoker: Average number of cigarettes/pipes per day  <5  5-9  10-14  15-24  >=25 | 4 (27)  0 (0)  5 (33)  5 (33)  1 (7) | 2 (13)  3 (20 4 (27)  2 (13)  4 (27) | 6 (20)  3 (10)  9 (30)  7 (23)  5 (17) |
| Alcohol consumption – no. (%)  Never  Less than 1 unit/week  Current drinker | 12 (33)  14 (39)  10 (28) | 14 (40)  13 (37)  8 (23) | 26 (37)  27 (38)  18 (25) |
| Physical activity level – no. (%)  Low  Moderate  Vigorous | 24 (67)  11 (31)  1 (3) | 25 (71)  10 (29)  0 (0) | 49 (69)  21 (30)  1 (1) |
| **Other Clinical Characteristics** | | | |
| Co-morbidities – no. (%)  Malignancy  Hypertension  Stroke  Myocardial infarction  High cholesterol  Angina  Diabetes  Deep vein thrombosis | 4 (11)  15 (42)  5 (14)  3 (8)  9 (25)  3 (8)  12 (33)  12 (33) | 3 (9)  19 (54)  3 (9)  4 (11)  7 (20)  2 (6)  4 (11)  11 (31) | 7 (10)  34 (48)  8 (11)  7 (10)  16 (23)  5 (7)  16 (23)  23 (32) |
| Current medications – no. (%)  Antiplatelets  Steroids  Pentoxifylline  Statins  Anticoagulants  Antibiotics  Oral contraceptive pills (women only)  Hormone replacement therapy (women only) | 6 (17)  4 (11)  1 (3)  14 (39)  15 (42)  6 (17)  0 (0)  0 (0) | 3 (9)  6 (17)  0 (0)  12 (34)  14 (40)  3 (9)  0 (0)  0 (0) | 9 (13)  10 (14)  1 (1)  26 (37)  29 (41)  9 (13)  0 (0)  0 (0) |
| Previous varicose vein surgery/intervention on reference leg – no. (%) | 23 (64) | 21 (60) | 44 (62) |
| Type of surgery/intervention – no. (%)  GSV surgery  SSV surgery  UGFS  Laser ablation  Radiofrequency ablation  Phlebectomy  Unknown  Other | 5 (22)  3 (13)  7 (30)  4 (17)  6 (17)  0 (0)  1 (4)  5 (22) | 6 (29)  2 (10)  8 (38)  3 (14)  3 (14)  1 (5)  3 (14)  2 (10) | 11 (25)  5 (11)  15 (34)  7 (16)  9 (20)  1 (2)  4 (9)  7 (16) |

**Table S2. Healed index ulcer at 12 months after randomisation**


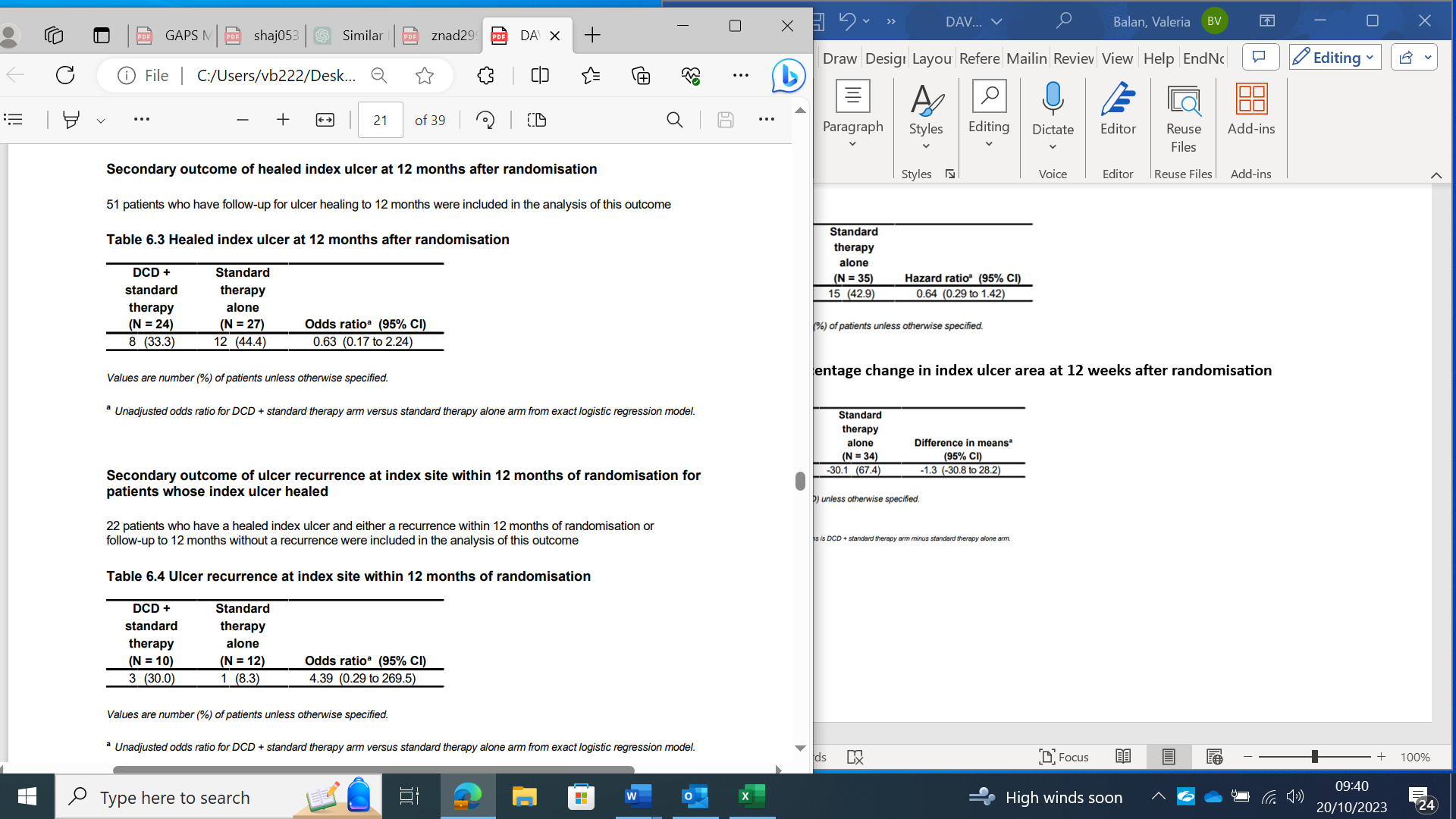


**Table S3. Percentage change in index ulcer area at 12 weeks after randomisation**


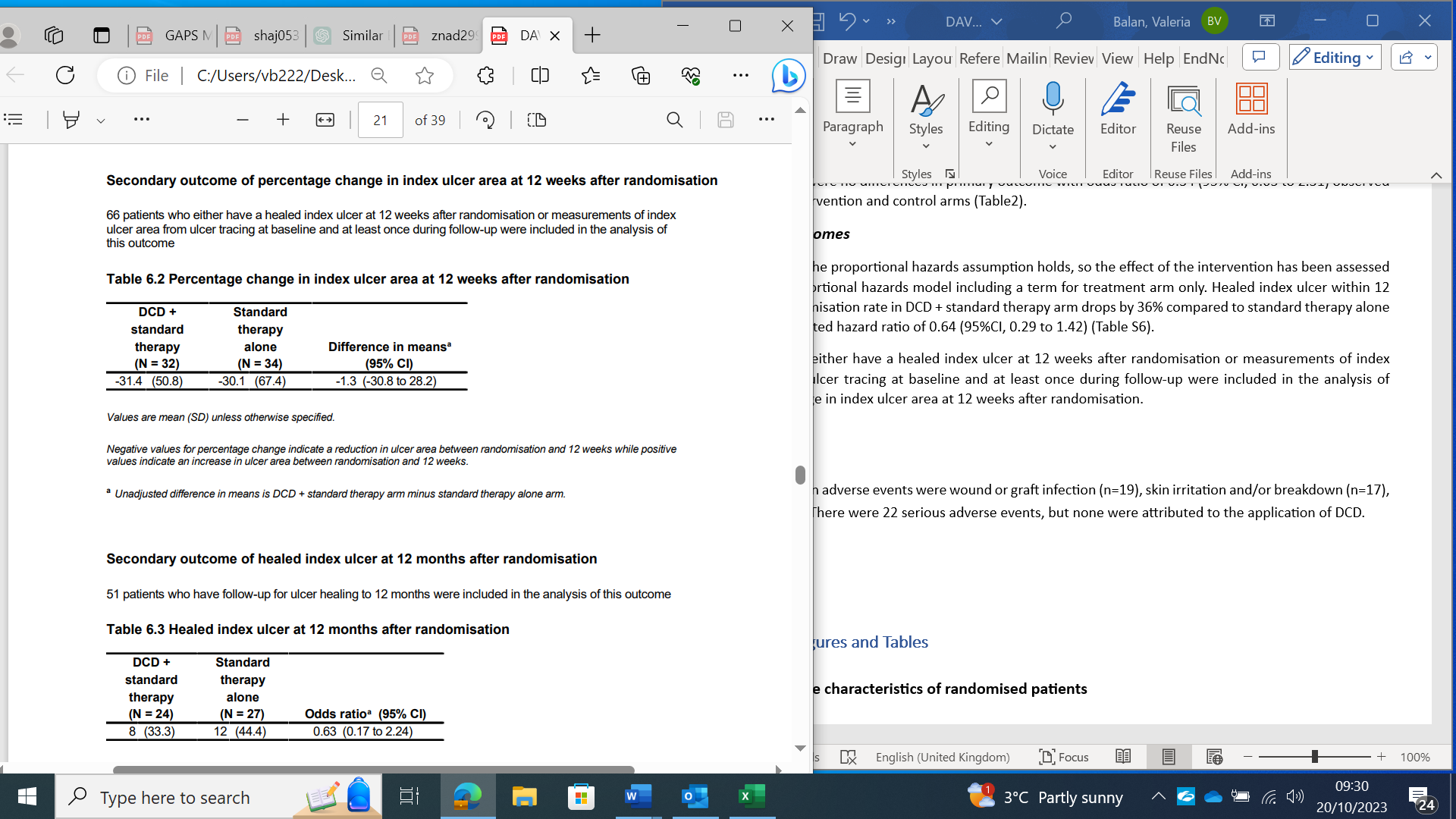


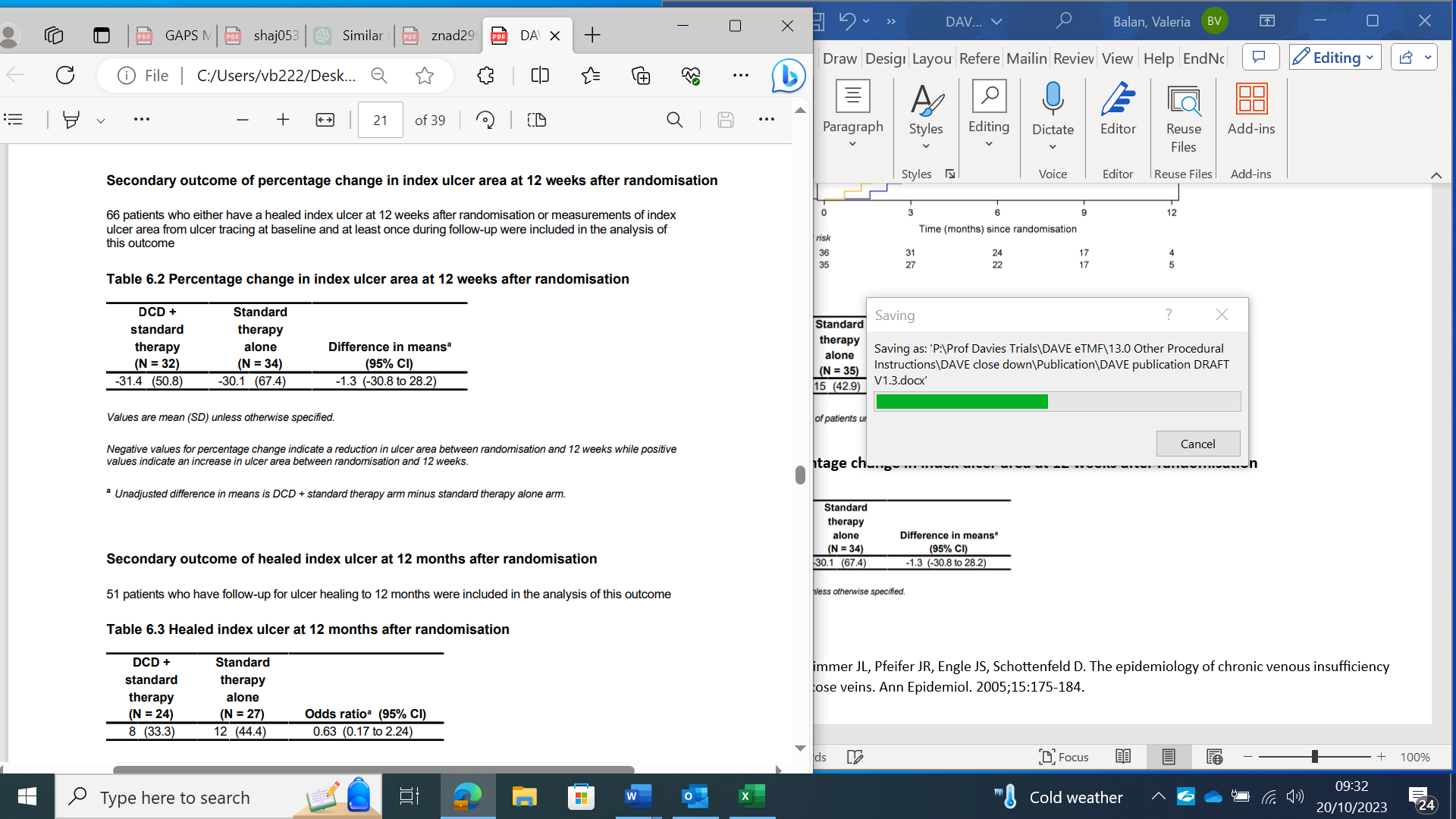


**Table S4. Ulcer recurrence at index site within 12 months of randomisation**
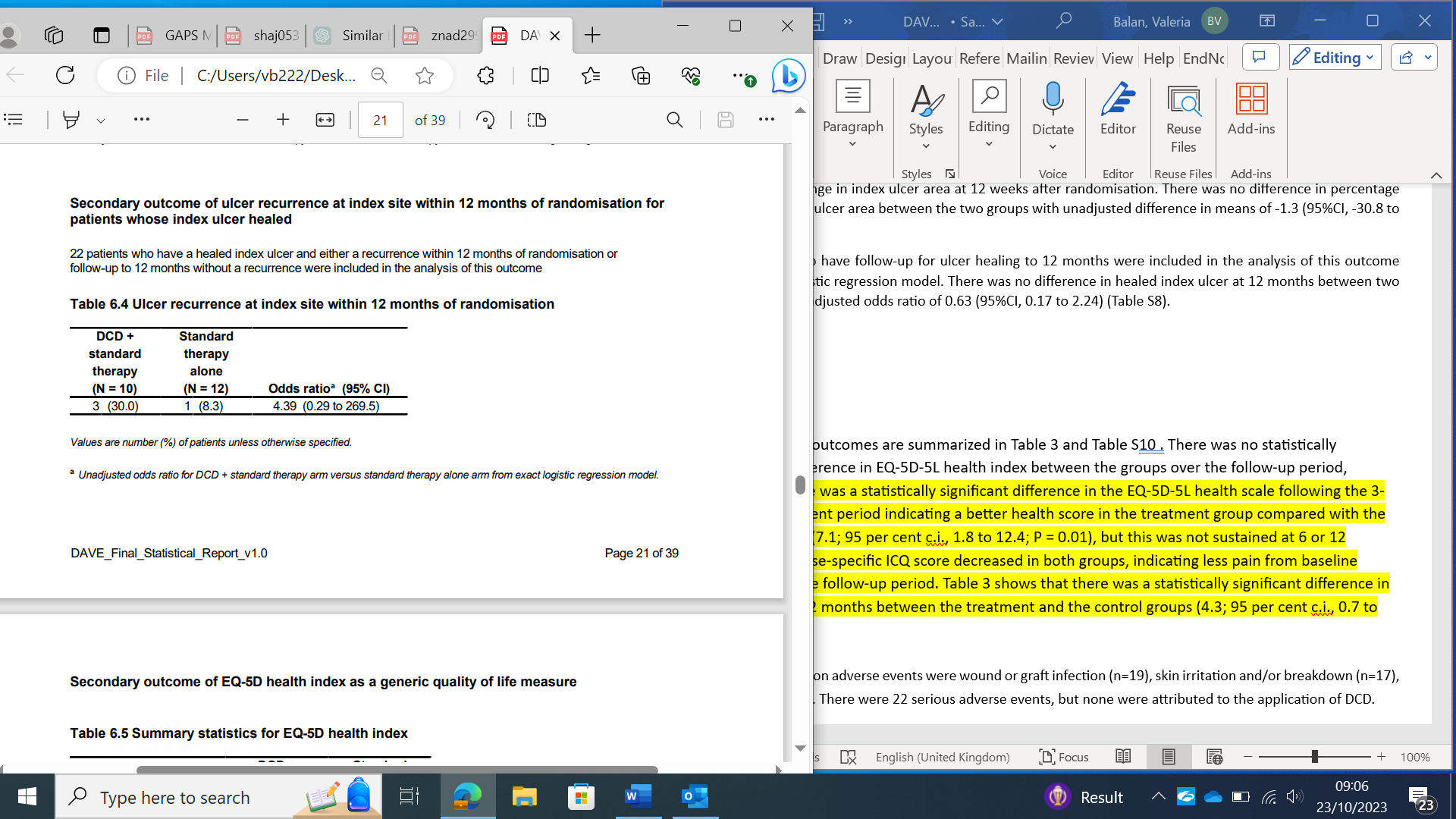


**Table S5. Summary statistics for generic and disease-specific health-related quality of life measures**

|  | **EQ-5D-5L Health Index** | | **EQ-5D-5L Visual Analogue Scale** | | **Charing Cross Venous Ulcer Questionnaire** | |
| --- | --- | --- | --- | --- | --- | --- |
| **Timepoint** | **DCD (n = 36)** | **SOC (n = 35)** | **DCD (n = 36)** | **SOC (n = 35)** | **DCD (n = 36)** | **SOC (n = 35)** |
| **Baseline**  N (# missing)  Mean (SD) | 36 (0)  0.478 (0.328) | 35 (0)  0.512 (0.293) | 36 (0)  67.6 (23.3) | 35 (0)  62.5 (21.9) | 36 (0)  59.7 (17.7) | 35 (0)  61.0 (17.3) |
| **12 weeks**  N (# missing)  Mean (SD) | 32 (4)  0.592 (0.285) | 32 (3)  0.507 (0.301) | 32 (4)  66.0 (20.9) | 32 (3)  67.3 (21.0) | 32 (4)  51.7 (17.8) | 32 (3)  56.4 (17.8) |
| **6 months**  N (# missing)  Mean (SD) | 30 (6)  0.539 (0.351) | 30 (5)  0.557 (0.276) | 30 (6)  64.2 (24.5) | 30 (5)  65.1 (19.5) | 30 (6)  48.5 (20.4) | 30 (5)  55.7 (20.0) |
| **12 months**  N (# missing)  Mean (SD) | 24 (12)  0.505 (0.315) | 26 (9)  0.658 (0.224) | 24 (12)  62.1 (24.1) | 26 (9)  69.7 (18.5) | 24 (12)  49.5 (19.5) | 26 (9)  44.8 (19.4) |

Health index values were calculated from responses by patients to the five questions on EQ-5D-5L questionnaire using crosswalk algorithm and Dolan value set for the UK, and values can range from -0.594 to 1 with higher values indicating a better quality of life.

Health scale values are from completion by patients of the visual analogue scale on EQ-5D-5L questionnaire, and values can range from 0 to 100 with higher values indicating a better quality of life.

Scores were calculated from responses by patients to the twenty one questions on Charing Cross Venous Ulcer Questionnaire, and scores can range from 19 to 94 with higher scores indicating a worse quality of life.

**Table S6. Difference in means at each timepoint for generic and disease-specific health-related quality of life measures**

|  | **EQ-5D-5L Health Index** | | | **EQ-5D-5L Visual Analogue Scale** | | | **Charing Cross Venous Ulcer Questionnaire** | | |
| --- | --- | --- | --- | --- | --- | --- | --- | --- | --- |
| **Timepoint** | **DCD (n = 33)** | **SOC (n = 32)** | **Difference in means (95% CI)** | **DCD (n = 33)** | **SOC (n = 32)** | **Difference in means (95% CI)** | **DCD (n = 33)** | **SOC (n = 32)** | **Difference in means (95% CI)** |
| **12 weeks** | 0.594 (0.0389) | 0.497 (0.0390) | 0.097 (-0.013 to 0.207) | 63.7 (3.21) | 68.2 (3.20) | -4.5 (-13.5 to 4.6) | 53.1 (2.35) | 55.3 (2.35) | -2.2 (-8.8 to 4.4) |
| **6 months** | 0.547 (0.0467) | 0.560 (0.0467) | -0.013 (-0.145 to 0.119) | 63.2 (3.37) | 66.7 (3.37) | -3.5 (-13.1 to 6.0) | 49.5 (2.86) | 54.2 (2.84) | -4.6 (-12.7 to 3.4) |
| **12 months** | 0.530 (0.0389) | 0.635 (0.0375) | -0.105 (-0.214 to 0.003) | 61.6 (3.86) | 70.7 (3.72) | -9.1 (-19.9 to 1.6) | 49.9 (3.14) | 45.8 (3.04) | 4.1 (-4.7 to 12.8) |

Values are estimated mean (standard error of mean) in each treatment arm and difference in estimated means (95% CI) from repeated measures linear mixed model adjusting for measurements at baseline and allowing for correlations between the repeated measurements during follow-up.

**Table S7. Serious and non-serious adverse events**


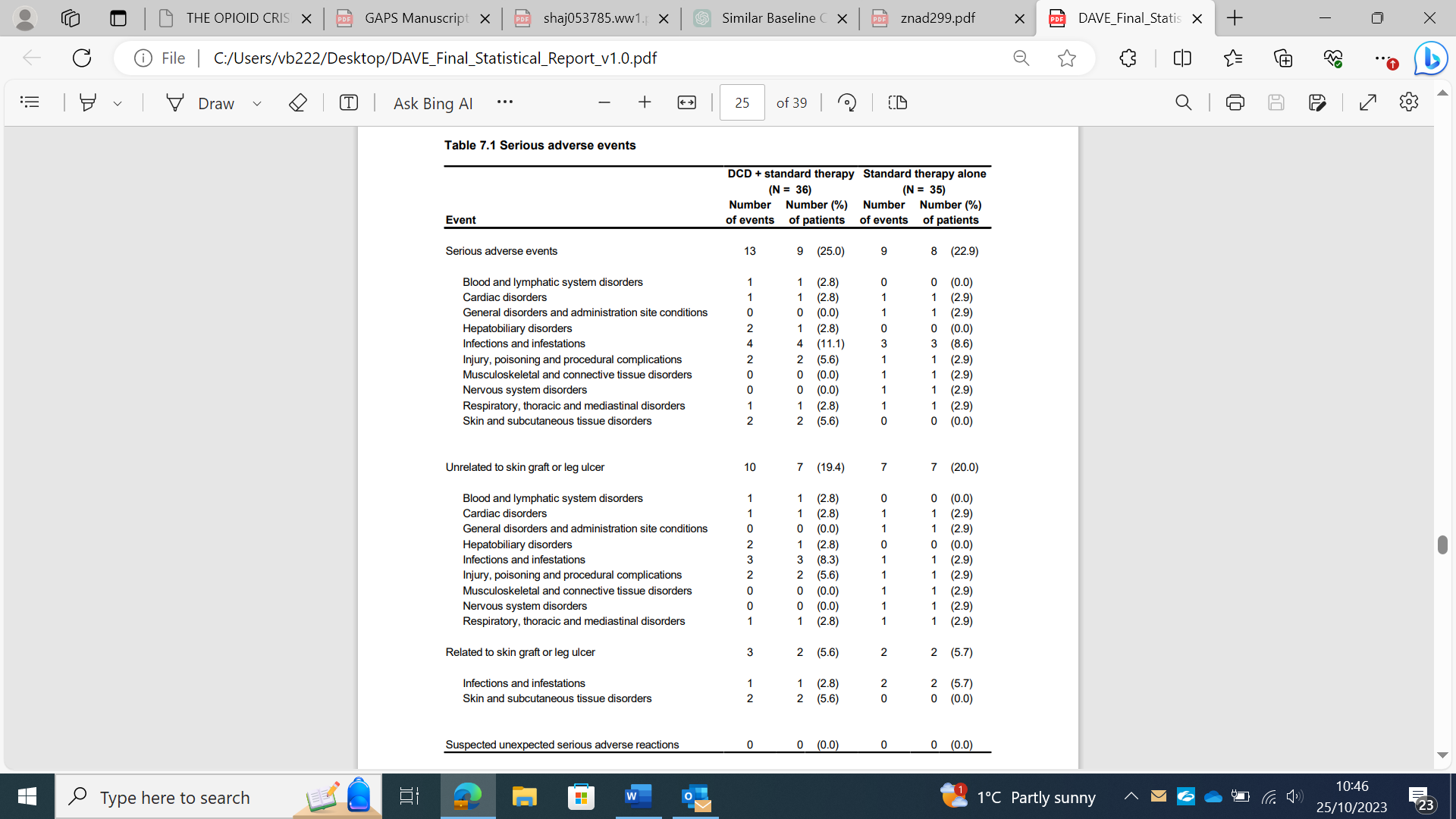


**Table S8. Non-serious adverse events related to skin graft or leg ulcer**


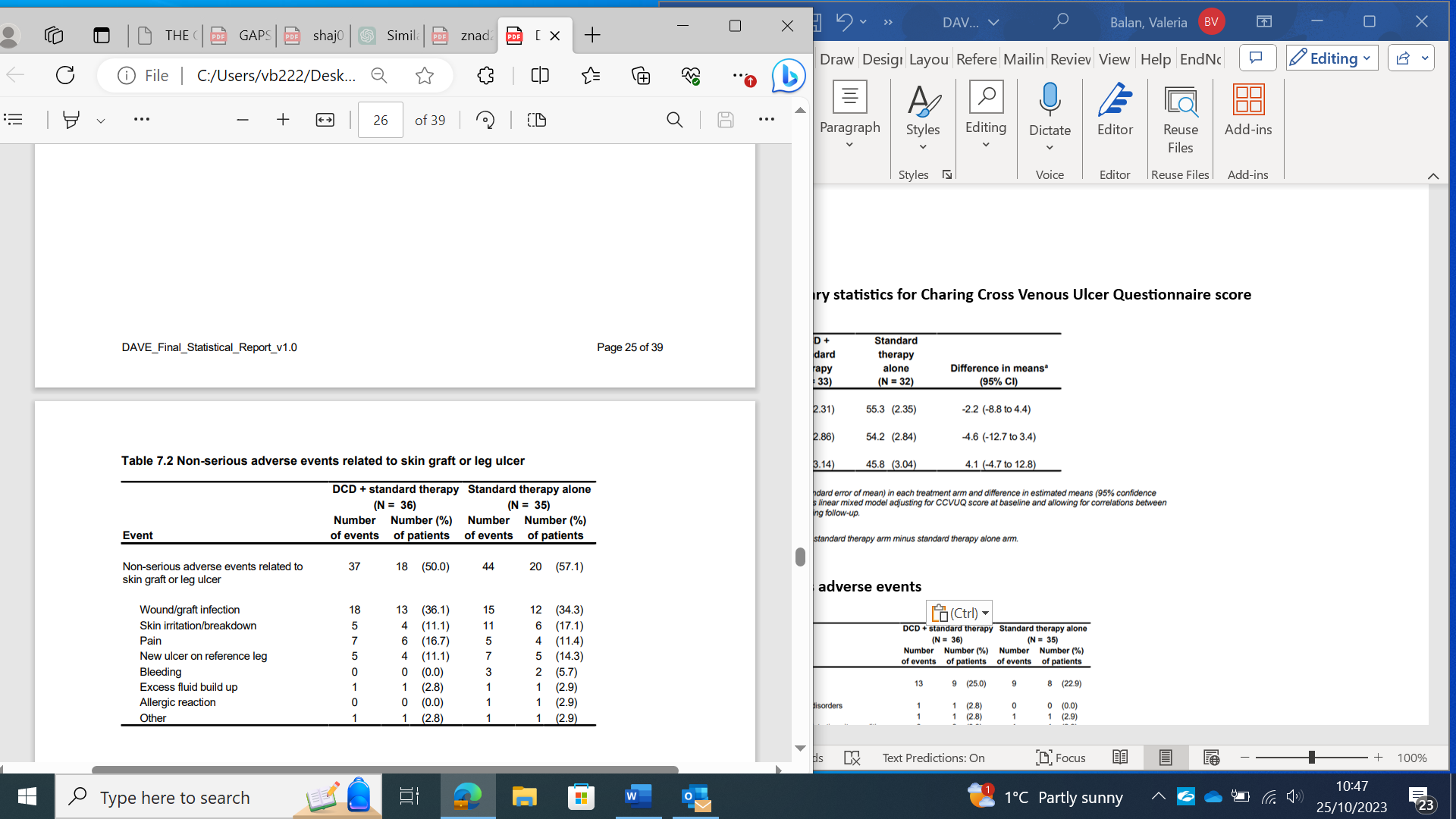

Supplement: znae330_Supplementary_Data [file znae330_supplementary_data.docx]
